# A Nighttime Vehicle Detection Method with Attentive GAN for Accurate Classification and Regression

**DOI:** 10.3390/e23111490

**Published:** 2021-11-11

**Authors:** Yan Liu, Tiantian Qiu, Jingwen Wang, Wenting Qi

**Affiliations:** School of Computer and Communication Engineering, Zhengzhou University of Light Industry, Zhengzhou 450066, China; qiutiantian@zzuli.edu.cn (T.Q.); 2015070@zzuli.deu.cn (J.W.); 331907040415@zzuli.edu.cn (W.Q.)

**Keywords:** nighttime vehicle detection, attentive GAN, multiple local regression, improved RoI pooling

## Abstract

Vehicle detection plays a vital role in the design of Automatic Driving System (ADS), which has achieved remarkable improvements in recent years. However, vehicle detection in night scenes still has considerable challenges for the reason that the vehicle features are not obvious and are easily affected by complex road lighting or lights from vehicles. In this paper, a high-accuracy vehicle detection algorithm is proposed to detect vehicles in night scenes. Firstly, an improved Generative Adversarial Network (GAN), named Attentive GAN, is used to enhance the vehicle features of nighttime images. Then, with the purpose of achieving a higher detection accuracy, a multiple local regression is employed in the regression branch, which predicts multiple bounding box offsets. An improved Region of Interest (RoI) pooling method is used to get distinguishing features in a classification branch based on Faster Region-based Convolutional Neural Network (R-CNN). Cross entropy loss is introduced to improve the accuracy of classification branch. The proposed method is examined with the proposed dataset, which is composed of the selected nighttime images from BDD-100k dataset (Berkeley Diverse Driving Database, including 100,000 images). Compared with a series of state-of-the-art detectors, the experiments demonstrate that the proposed algorithm can effectively contribute to vehicle detection accuracy in nighttime.

## 1. Introduction

With the continuous increase in the number of vehicles on the road, traffic accidents occur frequently, and traffic safety problems are becoming more and more serious. With the aim of fundamentally alleviating road traffic pressure and reducing the occurrence of traffic accidents, Intelligent Transportation Systems (ITS) [1] was proposed. As a part of the ITS, the intelligent driving system [2] uses advanced information technology to provide road driving assistance to the driver to make up for the lack of driver response capabilities. Vehicle detection is the most critical part of ITS, which provides important support for solving traffic problems. A variety of important road traffic information can be obtained through vehicle detection process, such as vehicle location, vehicle type, and distance between vehicles, which can provide a basis for judging vehicle driving and help connectivity in urban road scenes [3,4]. Vehicle detection is an important part of Automatic Driving System (ADS), which has achieved great improvements in recent years. The current research on vehicle detection has very important theoretical significance and application value.

Vehicle detection usually detects vehicles accurately by extracting vehicle features. The vehicle characteristics are more obvious, and vehicle detection is more accurate in the daytime. However, in night conditions, vehicle detection experiences difficulties, owing to unclear vehicle features and the complicated lighting environment. In the night environment, the visibility of the road environment becomes poor, while the road condition information obtained by the driver is often inaccurate, which makes the situation very prone to traffic accidents. In addition, when driving at night, the driver simply visually perceives the surrounding situation. Lack of judgment on road information and deterioration of the front vision at night makes it difficult for drivers to distinguish the distance information of the vehicle. Therefore, the traffic accident rate in the night environment is high.

Many researchers [5,6,7,8,9,10] have worked on vehicle detection problems. Compared with traditional methods, deep learning methods have achieved excellent performances. Deep learning-based vehicle detection methods are adopted to handle the problem of vehicle detection. Unfortunately, these detection methods could function well in daytime vehicle detection and they may have less accuracy and bad performance in nighttime conditions. In nighttime conditions, the overall brightness is quite low and the detailed information of vehicles, such as the edges and shape features of vehicles, is not clear. Therefore, contrast and details must be improved at night to achieve higher detection accuracy.

To tackle the abovementioned problems on nighttime vehicle detection, a new method is proposed. Its main contributions are summarized as follows.

1. An improved GAN is used to acquire representative features, which is named Attentive GAN. U-Net with an attention module is used as the generator and the global and local discriminator is used to balance the local dark regions and overall dark area;
2. To get accurate target localization, a multiple local regression is employed in the regression branch, which predicts multiple bounding box offset;
3. For precise classification, an improved RoI pooling method is used in the classification branch which assigns different weights to different sampling points based on deformable RoI pooling.

## 2. Related Work

Vehicle detection and state prediction methods [11,12] are very important and suitable for all road sections. Therefore, researchers have put significant efforts on the design of a vehicle detection algorithm to improve the detection accuracy and help drivers gain road traffic information easily [13]. At present, vehicle detection methods [14,15,16] mainly involve two types: traditional vehicle detection methods and deep learning-based vehicle detection methods.

The method using vehicle light information as vehicle features to detect and recognize the acquired vehicle features is one of the most widely used traditional vehicle detection methods, which cannot eliminate the interference of road reflections and is inaccurate in detecting vehicles in two-way driving lanes [17]. Another traditional method is Deformable Parts Model (DPM) [18], which has strong robustness to the deformation of the target through the detection method of parts. Subsequently, Chen et al. [19] first used Gaussian filtering to enhance the contrast of the taillights, extracted the value of the C_r_ channel in the YC_b_C_r_ space, and selected the region of interest through an adaptive threshold, and then performed the fast Fourier transform, and used the AdaBoost classifier for training and classification. Kim J et al. [20] proposed improvements on the traditional HOG features and proposed πHOG features based on location information and light intensity. According to the πHOG feature, SVM [21], ELM [22], and KNN [23] were used to train and detect vehicles, respectively. Traditional detection methods need to extract artificial features which have strict requirements regarding the environment. The training of SVM classifiers requires prior knowledge of the characteristics of related vehicles, which is complicated in operation and difficult to apply to practical applications.

With the tremendous progress made in deep learning research, the use of convolutional neural network (CNN) [24,25] to solve image detection problems has become a major trend. Compared with conventional methods, CNN-based object detectors have gained significant improvements in vehicle detection. Deep learning-based vehicle detection approaches roughly fall into two main types of groups: one-stage detection approaches [26,27,28,29] and two-stage detection approaches [30,31,32,33]. The one-stage vehicle detection method does not need to choose candidate regions, but directly converts the classification and positions of the target into a regression problem. It is a vehicle detection method with a simple network structure and real-time performance. Two-stage detection approaches need to generate region proposals and use these proposals to detect objects, which have higher accuracy. Focusing on improving the performance of vehicle detection, a significant amount of research is conducted on the basis of basic vehicle detection methods. Huang et al. [34] proposed a model called M-YOLO which uses the lightweight network MobileNetv2 as the feature extraction backbone network. K-means algorithm was used to cluster the dataset. For fast vehicle detection, Hoanh Nguyen [35] proposed an improved framework based on Faster R-CNN [36]. MobileNet architecture [37] was applied to build the base network in the original Faster R-CNN [36] framework. Hu et al. [38] combined multifeatured fusion and convolutional neural network to the vehicle detection method.

However, the detection methods mentioned above can get excellent performance on common vehicle detection, which are not properly suitable for nighttime vehicle detection. To deal with the detection problems in nighttime, GAN [39,40,41] has shown excellent performance on image processing recently. There is a limited number of research that apply the GAN network to nighttime vehicle detection, and some of them have limitations. Lin et al. [42] proposed a GAN-based data augmenter named AugGAN to expand the dataset for improving the performance of the nighttime vehicle detector. However, the processed image obtained by this nighttime vehicle detection method is noisy and has distortion. Based on CycleGAN, Shao et al. [43] combined features of night and day, and proposed a multi-scale feature fusion to enhance night vehicle feature detection. However, small vehicles in the remote distance are always discarded. To tackle the abovementioned problems on nighttime vehicle detection, the proposed method is introduced. The proposed method can eliminate the interference of road reflections, which is a difficult part in traditional vehicle detection methods. In addition, the proposed method has remarkable performance in most complex conditions, even in extremely dark, occluded, and dazzling scenes.

## 3. Method

**Figure 1:**
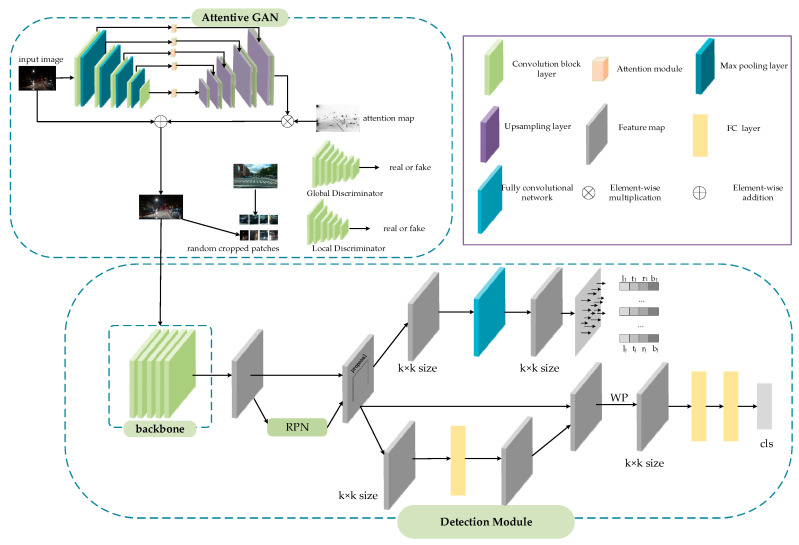
The overall framework of the proposed method.

In this part, the proposed method is described in detail. The overall framework of the proposed approach is depicted in Figure 1, which is based on the Faster R-CNN [44] framework. To eliminate the effect of weak environmental light or complex vehicle light at night, attentive GAN is introduced to get precise accuracy. We use multiple local regression, replacing the traditional box offset regression of Faster R-CNN, which will achieve more precise localization. In the classification module, an improved RoI pooling is used to accurately classify the vehicle, which extracts the features of different sub-regions of the candidate region, and then assigns adaptive weights to obtain discriminative features. In the following, the proposed method will be explained.

### 3.1. Attentive GAN Module

The Generative Adversarial Network (GAN) [39] is introduced into the method to improve the accuracy of nighttime vehicle detection, which contains a generator G and a discriminator D. An attentive U-Net [45] is used as the generator of GAN, while a global discriminator and local discriminator are used to improve the global light and enhance local regions.

#### 3.1.1. Attentive Generator

**Figure 2:**
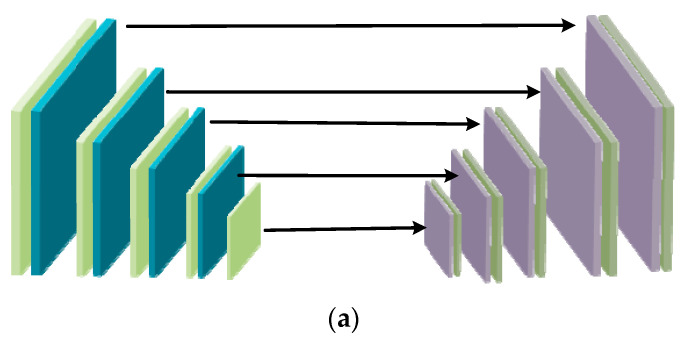

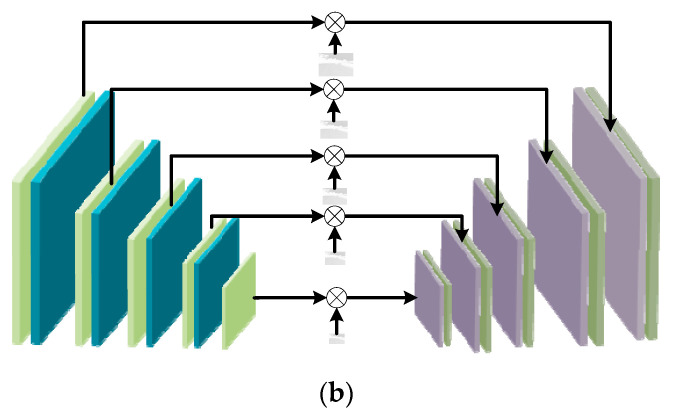
(**a**) The structure of U-Net; (**b**) the structure of attentive U-Net.

U-Net is used as the generator backbone for the reason that U-Net retains multi-scale context information. As shown in Figure 2a, U-Net is a U-shaped network which connects the encode layers to the decode layers. It helps information flow correctly from the encoder to the decoder. In order to deal with night image problems, an attention scheme is introduced into the GAN. Attention modules are added to each layer of encoder and decoder connections, which is shown in Figure 2b. With the purpose of balancing the dark regions and the bright regions in night image, *IC*, which is the illumination channel of the RGB image is normalized to [0, 1] and 1-*IC* is used as the attention map. The attention map is resized to different sizes, which can be used to multiply with the intermediate feature maps.

#### 3.1.2. Discriminator

Using the global discriminator alone cannot solve the problems of overexposure and underexposure in the local areas of the night image. Therefore, on the basis of the global discriminator, a local discriminator is added to solve the problems of overexposure and underexposure in local areas. PatchGAN, which retains a certain high resolution and high detail retention of image information is used in both the local and global discriminator. A local discriminator extracts patches randomly from the generator output and normal images, and learns to distinguish them between fake and real. The relativistic discriminator structure [46] which can help the generator produce more realistic images is used in a global discriminator. The function of relativistic discriminator [46] can be represented:

(1) $$D_{R_{a}}(x_{real},x_{fake})=sigmoid(C(x_{real})-E_{x_{fake}∼P_{fake}}[C(x_{fake})]),$$

(2) $$D_{R_{a}}(x_{fake},x_{real})=sigmoid(C(x_{fake})-E_{x_{real}∼P_{real}}[C(x_{real})]),$$

where *C* represents the discriminator network, $x_{real}$ denotes sampling from the real distribution and $x_{fake}$ represents sampling from the fake distribution. sigmoid is the sigmoid function. However, the sigmoid function is replaced by the least-square GAN (LSGAN) [47] loss in this relativistic discriminator structure. Therefore, the loss functions are:

(3) $$L_{D}^{G\mathrm{lobal}}=E_{x_{real}∼P_{real}}[{\left(D_{R_{a}}(x_{real},x_{fake})-1\right)}^{2}]+E_{x_{fake}∼P_{fake}}[(D_{R_{a}}{\left(x_{fake},x_{real}\right)}^{2}],\;$$

(4) $$L_{G}^{G\mathrm{lobal}}=E_{x_{fake}∼P_{fake}}[{\left(D_{R_{a}}(x_{fake},x_{real})-1\right)}^{2}]+E_{x_{real}∼P_{real}}[(D_{R_{a}}{\left(x_{real},x_{fake}\right)}^{2}].$$

In the local discriminator, the output image of generator and real image are all cropped into several patches. Original LSGAN is used as the local discriminator loss. The standard LSGAN [47] can be represented as:

(5) $$L_{D}^{\mathrm{local}}=E_{x_{real}∼P_{realpatches}}[{\left(D(x_{real})-1\right)}^{2}]+E_{x_{fake}∼P_{fakepatches}}[{\left(D(x_{fake})-0\right)}^{2}],$$

(6) $$L_{G}^{\mathrm{local}}=E_{x_{real}∼P_{fakepatches}}[{\left(D(x_{fake})-1\right)}^{2}].$$

#### 3.1.3. Training Loss of Attentive GAN Module

In traditional visual tasks, a pre-trained VGG is always used to represent the feature distance between the generator output and the ground truth. In the proposed method, the feature distance between input night image and its output image is constrained, which can preserve the image content features of themselves. For this purpose, self-feature preserving loss [48] is used and it can be computed as:

(7) $$L_{SFP}(I)=\frac{1}{W_{i,j}H_{i,j}}\sum_{x=1}^{W_{i,j}}\sum_{y=1}^{H_{i,j}}(T_{i,j}(I)-T_{i,j}(G(I)){)}^{2},$$

where I represents the input and G(I) represents the output. $W_{i,j}$ and $H_{i,j}$ are the dimensions of the feature maps. $T_{i,j}$ represents the feature map generated from the pre-trained VGG model. i is max pooling, while j indicates convolutional layers after *i*-th max pooling.

Furthermore, similar feature preserving loss, $L_{SFP}^{Local}$, is used in the cropped local patches of local discriminator. In addition, after each feature map, an instance normalization layer [49] is used. Then, feature maps send to $L_{SFP}^{Local}$ and $L_{SFP}$ with the aim of having steady training. Therefore, the training loss of attentive GAN module can be expressed as:

(8) $$Loss=L_{SFP}^{Local}+L_{SFP}^{Global}+L_{G}^{Local}+L_{G}^{Global}.$$

### 3.2. Vehicle Detection Module

**Figure 3:**
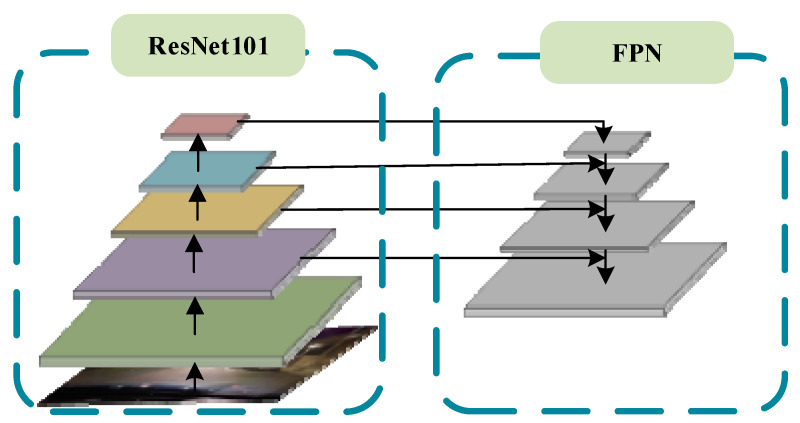
The backbone network: ResNet101 with FPN.

The vehicle detection module consists of four parts: the backbone network used for feature extraction, region proposal network (RPN), classification sub-network and regression sub-network. The image features are extracted by ResNet101 with FPN [50], and RPN is used to generate RoI proposals. In traditional Faster R-CNN, after getting proposals and feature maps, RoI Pooling [44] or RoI Align [51] is used to get fix-sized feature maps, which will send to some full connected layers to obtain the classification score and bounding box. Recently, several researchers have divided the classification and regression into two separate branches: the classification branch and the bounding box regression branch, which is beneficial to accuracy vehicle detection. Therefore, the classification branch is implemented by an improved RoI Pooling and the regression branch is realized by a multiple regression. In the proposed method, the backbone network of the vehicle detection module is ResNet101 with FPN, which can improve the model performance. As shown in Figure 3, ResNet101 with FPN is used to extract multi-scale features from the input night vehicle images. Compared with traditional backbone network of Faster R-CNN, ResNet101 with FPN can get deeper and more accurate feature information which will benefit to the later detection.

#### 3.2.1. Multiple Local Regression

In traditional Faster R-CNN, after getting proposals and feature maps, ROI pooling or RoI Align is used to generate the fix-sized (k × k) feature map within the proposal. Then, each feature map is sent to several fully connected layers, which will fall into two output layers: the bounding box regression branch and the classification branch. The branch of the bounding box regression is implemented by predicting the box offset ($t_{x}$,$t_{y}$, $t_{w}$,$t_{h}$) of each proposal to get accuracy bounding box. The offset can be represented by:

(9) $$\begin{array}{cc} t_{x}=(x_{G}-x_{P})/w_{P} & t_{y}=(y_{G}-y_{P})/h_{P}\\ t_{w}=\log(w_{G}/w_{P}) & t_{h}=\log(h_{G}/h_{P}) \end{array}\;,$$

where $x_{P}$, $y_{P}$, $w_{P}$, and $h_{P}$ are the center coordinates of proposal *P* and its width and height. Variables $x_{G}$, $y_{G}$, $w_{G}$, $h_{G}$ are the center coordinates of ground truth box *G* and its width and height.

**Figure 4:**
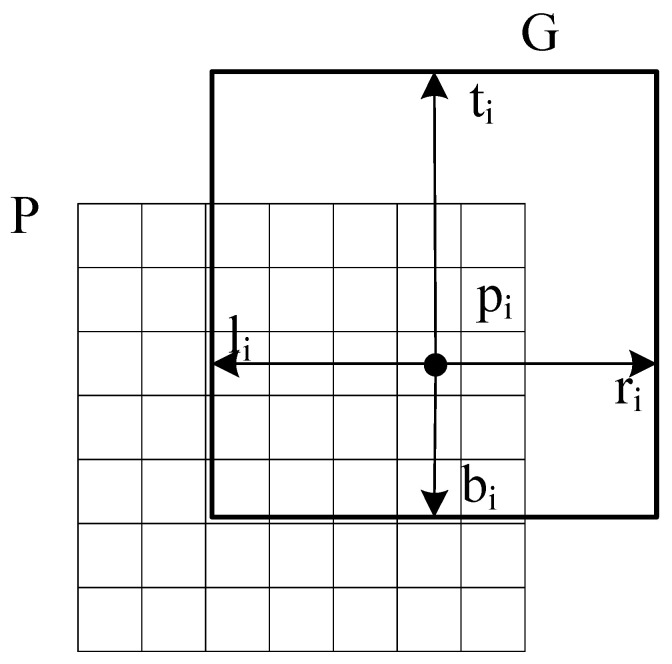
Multiple box offsets.

While in the proposed approach, unlike the traditional Faster R-CNN which predicts one box offsets, we selectively replace traditional bounding-box regression with multiple local regression. The proposed regression predicts multiple box offsets of proposal. In the multiple local regression branch, the fix-sized (k × k) feature map, which can get multiple local box offsets, is treated as k^2^ local features. As shown in Figure 1, the fix-sized (k × k) feature map is sent to a fully convolutional network to predict multiple box offsets. The multiple box offsets represent the distance of local feature *p_j_* at the position ($x_{j}$, $y_{j}$) to the bottom-right and top-left corner of the ground truth box as shown in Figure 4. The offsets ($\ell_{j}$, $t_{j}$, $r_{j}$, $b_{j}$) at position j can be calculated as follows:

(10) $$\begin{array}{cc} \ell_{j}=(x_{j}-x_{\ell })/w_{P} & t_{j}=(y_{j}-y_{t})/h_{P}\\ r_{j}=(x_{r}-x_{j})/w_{P} & b_{j}=(y_{b}-y_{j})/h_{P} \end{array}\;,$$

where ($x_{j}$, $y_{j}$) and ($x_{\ell }$, $y_{t}$) represent the bottom-right and the top-left of the ground truth box. $w_{P}$ and $h_{P}$ are the width and height of proposal *P*. These predicted multiple box offsets are applied to calculate the bottom-right and top-left corner positions of the predicted box. Then, boxes of local features prediction are average computed to get the final bounding box.

#### 3.2.2. Improved RoI Pooling

With the aim of getting more accurate vehicle classification results, the classification sub-branch is on the basis of the deformable RoI pooling [52] and makes some extensions to improve the classification. Similar to the standard deformable RoI pooling layers, the classification module also includes standard RoI pooling, a fully connected layer, and offsets.

**Figure 5:**
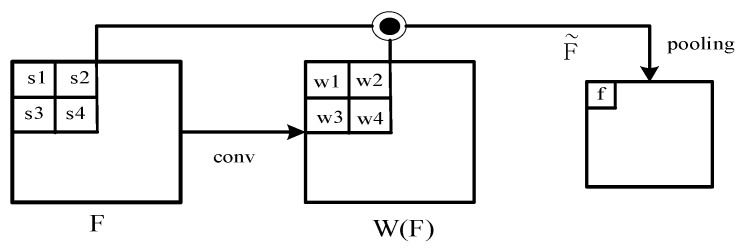
The weighted pooling (WP).

In deformable RoI pooling, RoI Align is used to generate fix-sized (k × k) pooled feature maps. Then, a fully connected layer is used to provide the normalized offsets of these feature maps. Different from the weighted strategy of the standard deformed RoI pooling, different weights are assigned to different sampling points obtained within the k × k feature maps. The weighted feature $\tilde{F}$ of the candidate proposal can be calculated by.

(11) $$\tilde{F}=W(F)⊙F$$

where F is the RoI Align feature in primal sampling points, W(F) is computed from *F* using the convolution operations. Furthermore, ⊙ is the Hadamard product. Several sampling points (s1, s2, s3, s4) and the computed weights (w1, w2, w3, w4). The weighted pooling (WP) process is shown in Figure 5. After $\tilde{F}$ is obtained, average pooling is performed, and finally the weighted RoI feature is obtained. Then, the feature is sent to fully connected layers to gain the final classification score.

#### 3.2.3. The Loss of Vehicle Detection Module

In vehicle detection module, the loss mainly contains two parts: the loss of regression and the loss of classification. Regression loss contains two components: RPN regression loss and the loss of multiple regression branch. Smooth L1 loss is chosen as RPN regression loss, which will improve the robustness of regression process. $S_{L1}$ loss can be expressed as:

(12) $$S_{L1}(x)=\{\begin{array}{cc} 0.5x^{2} & ,∥x∥\;<\;1\\ ∥x∥\;-\;0.5 & ,∥x∥\;\geq \;1 \end{array}\;,$$

where $x=p_{i}-p_{i}^{*}$. $p_{i}$ is a vector representing the four parameterized coordinates of the predicted bounding box and $p_{i}^{*}$ is the coordinate vector of ground truth box corresponding to positive anchor. IoULoss, which trains the location information as a whole, is used as the loss of multiple regression branch. Compared with smooth L1 loss, IoULoss contributes to more accurate training results, which is quite important for the final location. IoULoss can be calculated as:

(13) $$IoU=-\ln\frac{Inter\sec tion(P,G)}{Union(P,G)},\;$$

where P represents the bounding box prediction and G indicates the bounding box ground truth.

The classification loss is also composed of the RPN classification loss and the loss of classification branch, and cross entropy loss is used. Cross entropy loss is represented as:

(14) $$H_{CE}=-\sum_{i}p_{i}^{*}\log p_{i},$$

where $p_{i}$ represents the true label value, and $p_{i}^{*}$ is the predicted value. In RPN classification process, cross entropy loss is a binary cross entropy loss. Different from the loss of RPN classification, the loss of classification branch is cross entropy loss of multi-classifications.

## 4. Experiments

### 4.1. Datasets and Implementation

**Figure 6:**
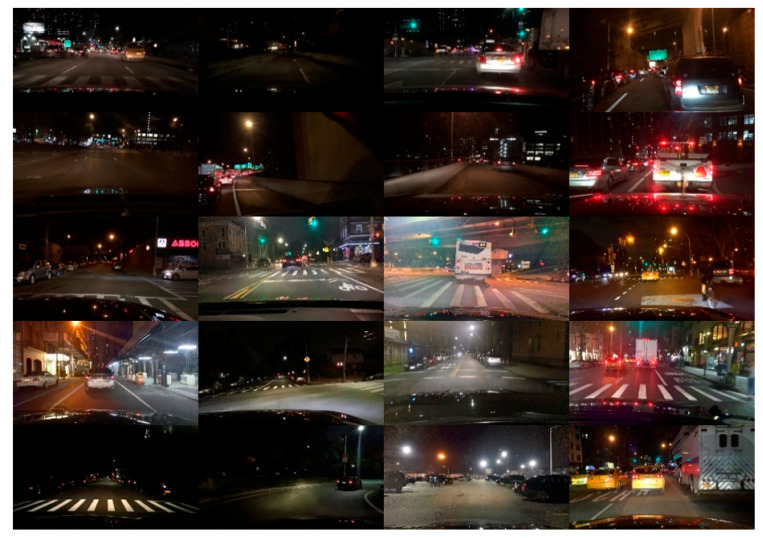
Nighttime vehicle dataset.

In order to demonstrate the effectiveness of the proposed method, this paper conducts experiments on the selected nighttime vehicle dataset. The selected dataset is composed of the partial nighttime images of Berkeley Deep Driving (BDD-100k) [53] dataset. The BDD-100k dataset is a naturalistic driving dataset, which contains 100k high resolution images. Various scene types are included, such as city streets, residential areas, and highways. Furthermore, it also includes different kinds of images at different times of the day. A total of 8075 nighttime images are randomly selected from BDD-100k dataset to evaluate the proposed method, 6075 images are selected to train the models and test, while 2000 images are included in the test set. A brief demonstration of the dataset is shown in Figure 6.

Images which are selected from BDD-100k are resized to 800 × 600. Our baseline models are all based on the framework of MMDetection, which is an object detection toolkit based on PyTorch. MMDetection contains dozens of state-of-the-art detectors, which make it easier for us to conduct our experiment. For precise detection, attentive GAN is used for more detailed image information. ResNet101 with FPN is chosen as the backbone of Faster R-CNN. To simplify hyperparameter tuning of algorithm [54,55], the Stochastic Gradient Descent (SGD) solver with momentum of 0.9, and the weight decay of 0.0001 on a single NVIDIA GeForce RTX 2080Ti GPU is used to optimize the network. The learning rate is initialized as 0.0025 for the first 20 epochs and decreased by 0.1 after 16th and 22th epochs.

### 4.2. The Results of Comparisons

We quantitatively analyze the experimental results of the proposed method on the selected BDD-100k dataset with state-of-the-art detection approaches, including two-stage detection approaches of Faster R-CNN, Cascade R-CNN [56], Mask R-CNN [51], and one-stage detection approaches of RetinaNet [57], and SSD [58]. These detection models are evaluated with the COCO metrics, including average precision (AP) over Intersection over Union (IoU) thresholds from 0.5 to 0.95, AP_50_ (IoU threshold over 0.5), AP_75_ (IoU threshold over 0.75). Furthermore, AP_S_, AP_M,_ and AP_L_ represent the results on small, medium, and large scales, respectively. As presented in Table 1, the proposed method obtains the greatest performance.

As shown in Table 1, compared with two-stage detection approaches, one-stage detection approaches really have poor detection accuracy. SSD gets the least AP value and it has bad performance under different vehicle sizes compared with other detection approaches. RetinaNet, another one-stage detection approach, shows an improvement compared with SSD. Two-stage detection approaches show more significant improvement in detection accuracy compared with one-stage detection approaches. However, the most accurate result is made by the proposed method. As shown in Table 1, the value of AP_S_ is quite lower than others, which reflects the problem that small target vehicles are not easy to detect. Compared with other approaches, the proposed method has notable AP_S_ gains, which indicates that proposed method has better performance in small target vehicles.

### 4.3. Qualitative Analysis

**Figure 7:**
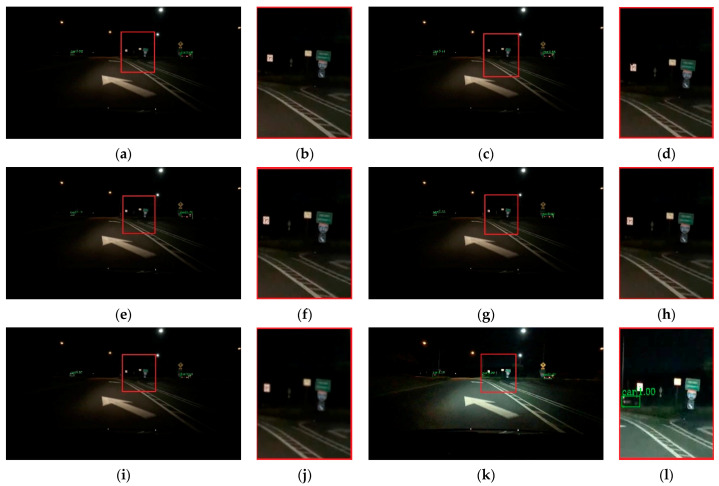
Detection results of dark scene. (**a**) faster R-CNN, (**c**) Cascade R-CNN, (**e**) Mask R-CNN, (**g**) RetinaNet, (**i**) SSD, (**k**) ours. (**b**,**d**,**f**,**h**,**j**,**l**) represent the red area of (**a**,**c**,**e**,**g**,**i**,**k**) respectively.

**Figure 8:**
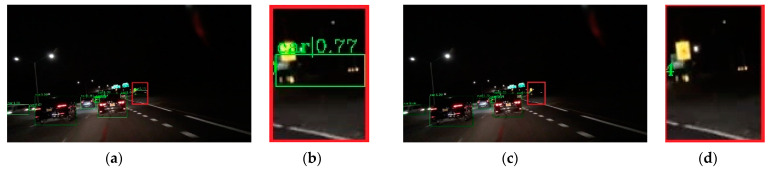

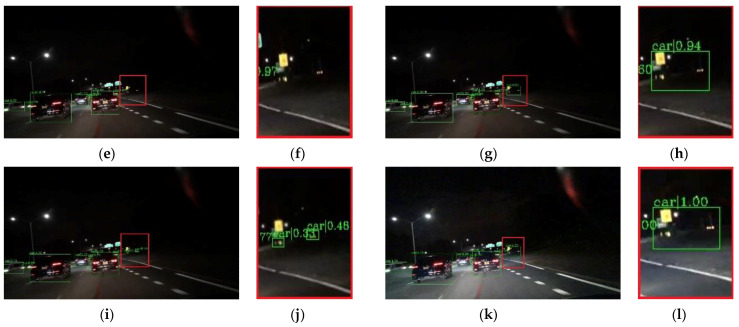
Detection results of normal night scene. (**a**) faster R-CNN, (**c**) Cascade R-CNN, (**e**) Mask R-CNN, (**g**) RetinaNet, (**i**) SSD, (**k**) ours. (**b**,**d**,**f**,**h**,**j**,**l**) represent the red area of (**a**,**c**,**e**,**g**,**i**,**k**) respectively.

**Figure 9:**
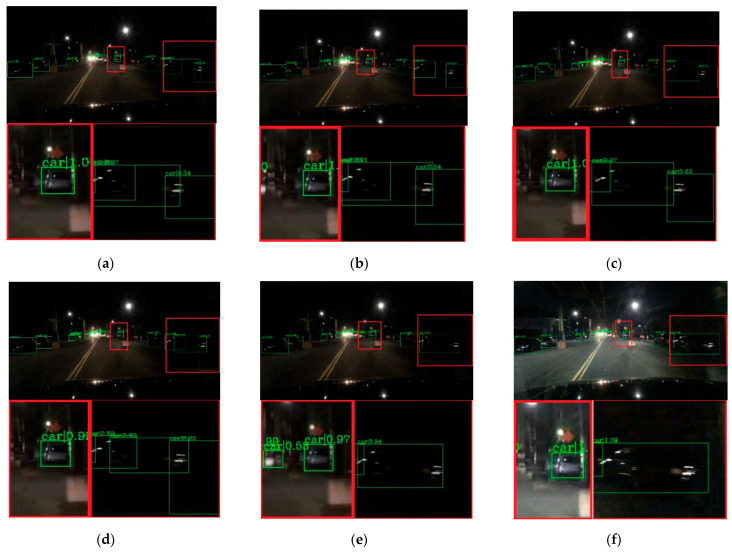
Detection results of partial dark and partial dazzling scene. The first line is the detection result image, and the second line is the red rectangle information in the corresponding detection result image (**a**) faster R-CNN, (**b**) Cascade R-CNN, (**c**) Mask R-CNN, (**d**) RetinaNet, (**e**) SSD, (**f**) ours.

**Figure 10:**
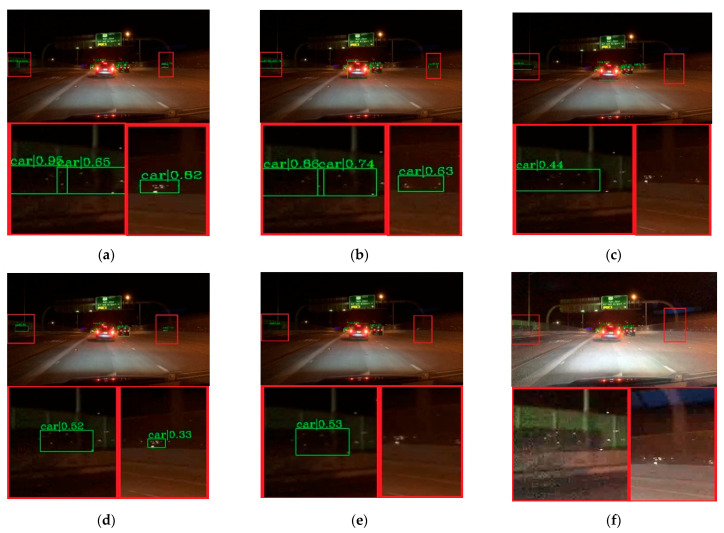
Detection result of the viaduct scene. The first line is the detection result image, and the second line is the red rectangle information in the corresponding detection result image (**a**) faster R-CNN, (**b**) Cascade R-CNN, (**c**) Mask R-CNN, (**d**) RetinaNet, (**e**) SSD, (**f**) ours.

**Figure 11:**
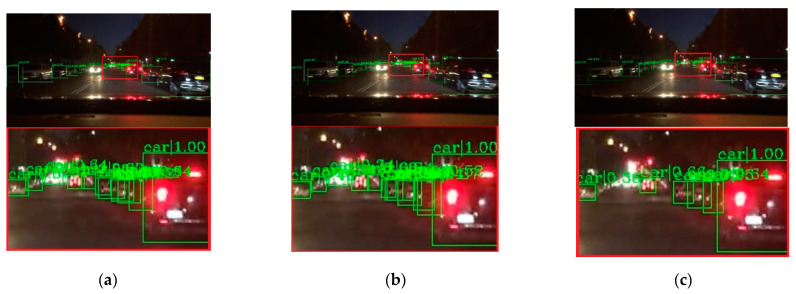

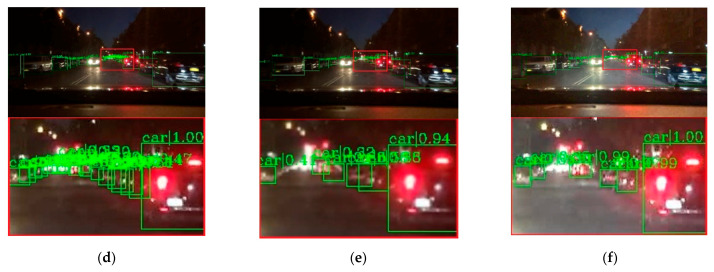
Detection results of dazzling and the partly occluded scene. The first line is the detection result image, and the second line is the red rectangle information in the corresponding detection result image (**a**) faster R-CNN, (**b**) Cascade R-CNN, (**c**) Mask R-CNN, (**d**) RetinaNet, (**e**) SSD, (**f**) ours.

To verify the effectiveness of the proposed method, visual comparisons are performed on the selected BDD-100k dataset. We choose some representative scene, and the detection results are presented in Figure 7, Figure 8, Figure 9, Figure 10 and Figure 11. The green rectangles in the resulting image represent the object location and sizes of the vehicles detected by the specific method. The red rectangles show the detailed information of the specific location.

As shown in Figure 7, the image shows low brightness and the vehicles features are not obvious, which will be a challenge for vehicle detection. All detectors show their detective performance with different accuracy. As the red rectangle shows, there is a car which is hardly recognized. Other state-of-the-art detectors have missed detections, while our proposed method can detect and achieves accurate results. Vehicle detection under low brightness has great importance, which can help drivers make appropriate choices. Even in normal scenes that are not too dark, missed detection problems still occur at a distance and an example is shown in Figure 8. It is obvious that the missed vehicle at a distance is difficult to be recognized by Cascade R-CNN and Mask R-CNN. Mask R-CNN missed detections, while SSD made error detection. The proposed method can recognize the difficult vehicle and perform better detection performance. In addition, the surrounding lights can make it difficult to detect vehicles correctly. In Figure 9, partial dark and partial dazzling scenes are a challenge for the detection approaches. It is obvious that SSD has made an error detection at a partial dazzling region, while the other detection approaches give the right detection result. In a partial dark area, only the proposed method and SSD give the right detection results. However, the proposed method shows higher accuracy. As presented in Figure 10, the interference from lights beside the viaduct causes incorrect detection results. RetinaNet, Faster R-CNN, and Cascade R-CNN make incorrect detection results on both sides of the viaduct, while Mask R-CNN and SSD make incorrect detection results on the left side of the viaduct. The proposed method shows the best performance, which only detects correct objects and has correct bounding box localizations. As shown in Figure 11, there are more error bounding box localizations in the detection result of the detection approaches because of the occlusion and interferential light. More right boxes are produced by the proposed method and more vehicles are detected. These results show that the proposed method achieves remarkable performance in the most complex conditions, even in extremely dark, occluded, and dazzling conditions.

### 4.4. Ablation Study

An ablation experiment is conducted on the selected dataset to examine the contributions of different components to the overall network. All experiments use ResNet101 with FPN as the backbone. The baseline is Faster R-CNN. We gradually apply the Attentive GAN, Multiple Local Regression and Improved ROI Pooling to the baseline and present the performance in Table 2.

First, Attentive GAN is applied to the baseline network, which will be beneficial to obtain more distinct vehicle features. Clearer vehicle features will contribute to more accurate detection results. As shown in Table 2, the improvement over the baseline by adapting the Attentive GAN can be clearly observed. By enhancing the nighttime image features with the Attentive GAN, a 2.9% AP improvement is acquired, which illustrates the benefit of adding this component. Next, a Multiple Local Regression module is adopted to the method to get more accurate locations. It is observed that compared with the baseline, the value of AP is increased by 1.9%. Additionally, applying Improved RoI Pooling module to the baseline will get more discriminative features for classification. The detection result is shown in Table 2, a reasonable improvement of 0.9% AP is contributed. As analyzed above, different components all make their contributions to the improvement of the detection accuracy. One thing that is very noticeable is that applying the three modules to the baseline can achieve the best performance.

## 5. Conclusions

A nighttime vehicle detection method is introduced to obtain accurate vehicle detection in this work. Initially, Attentive GAN is proposed to improve the vehicle features, which contributes a lot to the accuracy of detection. Additionally, multiple local regression module is used to obtain more accurate object localization, while an improved RoI pooling module is used to generate precise classification and higher detection confidence score. By integrating the three modules, the proposed method can effectively detect vehicles with a small size and partial occlusion. The comparison detection results between the proposed and other state-of-the-art detection methods indicate that the proposed method has more competitive performance than others.

## Figures and Tables

**Table 1:** The comparisons of different detection approaches.

Method	Backbone	AP	AP_50_	AP_75_	AP_S_	AP_M_	AP_L_
Faster R-CNN	ResNet101 with FPN	35.7	58.2	38.8	8	32.1	56.2
Cascade R-CNN	ResNet101 with FPN	39.3	61.9	41.7	14	35.8	58.1
Mask R-CNN	ResNet101 with FPN	32.3	56.1	32.9	9.3	29	51.7
RetinaNet	ResNet101 with FPN	32.9	52.7	34.5	7.3	31.2	51
SSD	VGG16	29.9	53	30.2	5.2	26.8	48.8
Ours	ResNet101 with FPN	41.5	62.8	45.4	19.5	38	59.2

**Table 2:** Ablation results of each module on the selected nighttime vehicle dataset.

Baseline	Attentive GAN	Multiple Local Regression	Improved RoI Pooling	AP	AP_50_	AP_75_
✓				35.7	58.2	38.8
✓	✓			38.6	62	42.7
✓		✓		37.6	63	39.7
✓			✓	36.6	61	38.5
✓	✓	✓	✓	41.5	62.8	45.4

## Data Availability

Data is contained within the article.

## Abbreviations

The abbreviations in this article are as follows:

GAN	Generative Adversarial Network
RoI	Region of Interest
R-CNN	Region-Convolutional Neural Network
BDD	Berkeley Deep Driving
ITS	Intelligent Transportation Systems
ADS	Automatic Driving System
DPM	Deformable Parts Model
CNN	Convolutional Neural Network
LSGAN	Least-square Generative Adversarial Network
VGG	Visual Geometry Group Network
RPN	Region Proposal Network
FPN	Feature Pyramid Network
SSD	Single Shot MultiBox Detector
SGD	Stochastic Gradient Descent
IoU	Intersection over Union
